# Effects of Anethole on Meiotic Resumption and Oxidative Balance During in Vitro Maturation of Ovine Oocytes

**DOI:** 10.1111/rda.70296

**Published:** 2026-08-05

**Authors:** Anderson Nascimento de Andrade, Solano Dantas Martins, Alesandro Silva Ferreira, Naiza Arcângela Ribeiro de Sá, Jonathan Elias Rodrigues Martins, Daniela Ribeiro Alves, Selene Maia de Morais, José Henrique Leal‐Cardoso, Luciana Rocha Faustino, Vânia Marilande Cecatto, Valdevane Rocha Araújo

**Affiliations:** ^1^ Postgraduate Program in Physiological Sciences, Higher Institute of Biomedical Sciences State University of Ceará (UECE) Fortaleza Ceará Brazil; ^2^ Postgraduate Program in Biotechnology Parnaíba Delta Federal University (UFDPar) Parnaíba Piauí Brazil; ^3^ Laboratory of Research in Reproductive Physiology (FisioRep Lab), UFDPar Parnaíba Piauí Brazil; ^4^ International University of Afro‐Brazilian Lusofonia, Institute of Health Science Redenção Ceará Brazil; ^5^ Laboratory of Manipulation of Oocytes and Preantral Follicles, Veterinary Faculty State University of Ceará (UECE) Fortaleza Ceará Brazil; ^6^ Laboratory of Biochemistry and Gene Expression State University of Ceará (UECE) Fortaleza Ceará Brazil; ^7^ Natural Products Chemistry Laboratory, Animal Health Research Centre State University of Ceará (UECE) Fortaleza Ceará Brazil; ^8^ Histological Analysis and Processing Laboratory Federal University of Delta do Parnaíba (UFDPar) Parnaíba Piauí Brazil

**Keywords:** antioxidant potential, croton, oocyte quality, oxidative stress, sheep

## Abstract

The aim of this study was to evaluate the effect of anethole addition to the ovine oocyte in vitro maturation (IVM) medium on viability and chromatin configuration, intracellular production of reactive oxygen species (ROS), enzymatic activity of superoxide dismutase (SOD) and catalase (CAT), as well as the levels of reduced thiols in IVM medium, in addition to its antioxidant potential (DPPH and ABTS). Cumulus‐oocyte complexes (COCs) were randomly matured in IVM medium in the absence (control group, CTRL) or presence of anethole at concentrations of 30 (AN30), 300 (AN300), and 2000 μg/mL (AN2000). COCs were matured in groups under mineral oil at 39°C in a humidified atmosphere containing 5% CO_2_ in air for 24 h. At the end of IVM, part of the COCs was used to assess viability and chromatin configuration using Hoechst 33,342. The remaining COCs were used to assess intracellular ROS levels using the fluorescent probe H2DCFDA. Furthermore, the IVM medium was collected to evaluate antioxidant status (SOD, CAT, and reduced thiols). Finally, the antioxidant potential of different anethole concentrations was evaluated using DPPH and ABTS assays. It was observed that AN300 promoted higher meiotic resumption rates compared to the other treatments (*p* < 0.05) and a lower rate of oocyte degeneration compared to AN30 (*p* < 0.05). However, regarding the proportion of oocytes at MII, it was observed that only AN2000 was lower than AN300 (*p* < 0.05). Moreover, AN30 resulted in lower ROS levels compared to AN300 and AN2000 (*p* < 0.05). SOD activity was lower in AN2000 compared to the other treatments (*p* < 0.05). AN2000 also showed the highest antioxidant capacity in the DPPH and ABTS assays among the anethole concentrations, although it was lower than the control group. In conclusion, anethole supplementation during IVM positively affected oocyte meiotic progression in sheep.

## 1 Introduction

In vitro embryo production (IVEP) stands out as a biotechnological technique with the potential to accelerate and maximize reproduction, genetic improvement, and the sustainable development of sheep production. This technique consists of the steps of in vitro maturation (IVM), in vitro fertilization (IVF), and in vitro culture (IVC) of embryos. However, despite significant advances in recent years, ovine IVEP remains deficient, resulting in a blastocyst rate of 20%–30%, lower than in cattle (~40%; Paramio and Izquierdo 2014). Considering that IVM is the stage at which oocytes achieve meiotic competence, this is a determining factor in acquiring fertilizable, high‐quality oocytes for subsequent IVF (Rocha‐Frigoni et al. 2014).

The difficulty in producing good results in IVM is correlated with several factors, including a low density of cumulus cells surrounding the oocyte after collection (Araújo et al. 2010) and/or oxidative stress (Soto‐Heras and Paramio 2020), due to the excess of reactive oxygen species (ROS; Khazaei and Aghaz 2017). Therefore, the excessive production of ROS during IVM, resulting mainly from conditions intrinsic to the in vitro environment (pH, light, temperature, oxygen tension, and culture medium), in addition to excessive or inadequate handling of biological samples, may partially explain the limited results in embryo production (Torres‐Osorio et al. 2019). As a consequence of the high presence of ROS, structural and molecular alterations may occur (Agarwal et al. 2012) that result in low rates of oocyte nuclear maturation, reducing oocyte competence and causing cellular dysfunctions (Combelles et al. 2009). Thus, as a strategy to optimise IVM, the addition of natural antioxidants in the maturation medium aims to balance the excessive production of ROS (Khazaei and Aghaz 2017).

Antioxidants are molecules capable of inhibiting formation, eliminating, or stabilizing free radicals, or even acting in the process of repairing oxidative damage (Ribeiro et al. 2005). In recent years, several natural substances have been used in the maturation medium due to their antioxidant potential during IVM. Among them, anethole, or trans‐anethole, a secondary metabolite found in the essential oil of Croton zehntneri (Euphorbiaceae), a plant popularly known as ‘canela de cunhã’ and native to Northeastern Brazil (Sá et al. 2017; 2018), stands out. Anethole has been widely used in industry and traditional medicine (Sainio and Kanerva 1995) and its antioxidant potential was recently explored in ovarian follicles (Sá et al. 2017; da Silva et al. 2024; Sá et al. 2018; 2020, Silva et al. 2023), oocytes (Anjos et al. 2019; Sá et al. 2019; Janini et al. 2023; Conceição‐Santos et al. 2024), and embryos (Janini et al. 2023; Joo et al. 2023), achieving promising results. Despite this, these studies indicate that the effects of anethole vary depending on the concentration and the species under study.

Anethole was also able to stimulate endogenous antioxidant defence through the activity of in vivo superoxide dismutase (SOD) and glutathione enzymes (Giustarini et al. 2020) and in vitro catalase (da Silva et al. 2024), making it an excellent alternative for supplementing IVM media. Furthermore, to date, there are no reports in the literature on the effects of anethole on IVM in ovine oocytes. Therefore, the present study aimed to evaluate the effect of anethole at different concentrations added to the IVM medium of ovine oocytes on (1) oocyte maturation and viability rates, (2) ROS levels, (3) antioxidant and pro‐oxidant activity, as well as (4) the antioxidant potential of anethole.

## 2 Materials and Methods

This study was approved and performed under the guidelines of the Ethics Committee for Animal Use of the State University of Ceará under protocol number 03821216/2021.

### 2.1 Ovarian Source

Ovaries (*n* = 120) from mixed‐breed adult sheep were collected from a local slaughterhouse and washed once in 70% alcohol and twice in 0.9% NaCl (w/v). The ovaries were transported to the laboratory in 0.9% NaCl with antibiotics (100 IU/mL of penicillin and 100 μg/mL of streptomycin) at 33.5°C within 1 h.

### 2.2 Cumulus‐Oocyte Complexes (COC's) Recovery

In the laboratory, the ovaries were washed in transport solution (0.9% NaCl with antibiotics) and under sterile conditions, cumulus‐oocyte complexes (COCs) were recovered from the ovarian cortex by slicing using surgical blades (no. 24). COCs were selected in 0.9% saline solution plus 10% fetal bovine serum (FBS; Sigma Aldrich; F7524) under a stereomicroscope (SMZ 645 Nikon, Tokyo, Japan; × 100). Only COCs with homogeneous and non‐darkened cytoplasm, surrounded by at least two layers of compact cumulus cells and intact zona pellucida, were selected for IVM.

### 2.3 Experimental Design

To evaluate the effects of anethole on IVM of ovine oocytes, it was added to the IVM medium at three different concentrations (30, 300, or 2,000 μg/mL). The anethole concentrations for this study were chosen based on a previous study in bovine IVM (Sá et al. 2019). The medium used for IVM was TCM‐199 (Vitrocell; M0120) supplemented with 0.911 mM/L of pyruvic acid, 1% BSA, and 1 μg/mL of estradiol, designated TCM‐199+. COCs were randomly assigned to the following treatments: TCM‐199+ alone (control group‐CTRL) or TCM‐199+ supplemented with anethole at 30 (AN30), 300 (AN300), or 2,000 μg/mL (AN2000). The COCs were incubated for 24 h at 39°C under 5% CO_2_ in air in a five‐well plate (Nunc Cell Culture, Thermo Fisher Scientific, Waltham, Massachusetts, USA) containing 10 μL/COC of previously stabilized maturation medium. After IVM, the oocytes were evaluated for chromatin configuration and intracellular ROS levels using fluorescent probes. Furthermore, the IVM medium was collected for evaluation of activity of antioxidant enzymes and thiol content, as described below. The experiment was repeated five times, resulting in an average of 50 COCs per treatment.

### 2.4 Oocytes Viability and Meiotic Progression Assessment

After IVM, COCs were mechanically denuded by successive pipetting to assess oocyte viability and chromatin configuration by epifluorescence microscopy (40×, Eclipse 80i, Nikon, Tokyo, Japan). For this purpose, the oocytes were incubated in 10 μL/COC of phosphate‐buffered saline (PBS) containing 1% glutaraldehyde and 10 μM Hoechst 33,342 (emission at 483 nm) for 30 min. According to the chromatin configuration, the oocytes were classified into the stages: germinal vesicle (VG), germinal vesicle breakdown (GVBD), metaphase I (MI), metaphase II (MII), or degenerated (DEG) when the chromatin presented an abnormal configuration (Santos et al. 2019).

### 2.5 Total Intracellular ROS Production Assessment

Oocytes from different treatments (*n* = 10) were also evaluated for ROS production using the fluorescent probe 2′,7′‐dichlorodihydrofluorescein diacetate (H2DCFDA), as described by Fabbri et al. (2014). For this purpose, oocytes were incubated with 1 μL/mL of H2DCFDA for 30 min at 39°C. After incubation, oocytes were fixed in glutaraldehyde for 15 min at room temperature. Subsequently, the oocytes were mounted on slides and photographed under an epifluorescence microscope (20×, Eclipse 80i, Nikon, Tokyo, Japan). The images obtained were evaluated using ImageJ software, in which the average fluorescence intensity was calculated as previously described by Santos et al. (2019).

### 2.6 Biochemical Analyzes in Culture Medium

#### 2.6.1 Total Proteins (Bradford Method)

The protein concentration was determined using the Bradford method. This method uses Coomassie blue (Quick start/Bradford; Catalogue No. 500–0205; Bio‐Rad) to determine the total concentration of proteins in each extract sample. When it comes in contact with proteins, the Coomassie blue stain forms a complex and emits a blue luminescence. The absorbance is directly related to the protein concentration of the sample and was evaluated spectrophotometrically at a wavelength of 595_nm_. The total protein concentration in samples was determined using a standard curve constructed using bovine albumin as a standard (0, 2.5, 5, 10, 15, 25, 35 and 50 mg/mL), and was used to standardize the levels of thiol, CAT and SOD. Biochemical analyses were conducted according to Alves et al. (2026).

#### 2.6.2 Determination of Pro‐Oxidant Status Based on the Thiol Content

Total thiol content, 5,5′‐Dithiobis‐(2‐Nitrobenzoic Acid) (DTNB; Dynamics, D8130) was used as a reduced thiol index in the maturation medium after IVM. Thiol residues react with DTNB (10 mM), cleaving its disulfide bond and forming the 2‐nitro‐5‐thiobenzoate anion (NTB^2−^) at neutral pH. The NTB^2−^ concentration was quantified by measuring the absorbance at 412 nm using a spectrophotometer. The results were expressed as nmol of DTNB reduced per milligram of protein (Takahashi et al. 1978).

#### 2.6.3 Analysis of the Enzyme Catalase (CAT) Activity

CAT activity was measured by monitoring the consumption of hydrogen peroxide (H_2_O_2_) as a substrate at 240 nm (Aebi 1984). In a quartz cuvette, 50 μL of the IVM medium was added to 950 μL of solution of H_2_O_2_ (152 μL/mL; PH09717RA; Scientific Exodus) prepared in (PBS; pH 7.4) at room temperature. Every 30 s, the H_2_O_2_ consumption was measured in duplicate.

#### 2.6.4 Analysis of the Enzyme Superoxide Dismutase (SOD) Activity

SOD activity was measured with the inhibition of adrenaline auto‐oxidation (Bannister and Calabrese 1987). In a basic medium, the oxidation of adrenaline in the presence of CAT leads to the formation of the superoxide radical (O_2_^−^), which reacts with SOD, delaying (inhibiting) the oxidation of adrenaline. CAT solution (0.048 mg/mL; C9322; Sigma‐Aldrich, pH 10,2) and a sample of the IVM medium were added to the glycine buffer (Dynamics). Subsequently, adrenaline (0.218 mg/mL; E4260; Sigma‐Aldrich) was added to initiate the oxidation reaction. The oxidation was quantified by measuring the absorbance at 480 nm using a spectrophotometer every 10 s for a total of 180 s.

### 2.7 Evaluation of the Total Antioxidant Capacity of Anethole Through DPPH and ABTS Assays

Evaluation of antioxidant capacity by DPPH and ABTS free radical assays was measured in 96‐well flat‐bottom plates using the BIOTEK Elisa reader (model ELX 800, software Gen5 V2.04.1100), according to the methodology described by Becker et al. (2019), with some modifications (Silva et al. 2022). For DPPH (2,2‐diphenyl‐1‐picrylhydrazyl) analysis, 180 μL of methanolic DPPH solution and 20 μL of culture medium were added to each well. For both assays (ABTS and DPPH), the sample dilutions and 45 positive standards used in the quantitative evaluations of the microplates started from a stock solution with a concentration of 20,000 to 0.78 mg/mL. For the ABTS (2,2‐azino‐bis‐3‐ ethylbenzothiazoline‐6‐sulfonic acid) method, the ABTS solution (7 mM, 5 mL) was mixed with 88 mL of potassium persulfate (140 mM). The mixture was stirred and kept in the dark at room temperature for 16 h. Then, 1 mL of this solution was added to 99 mL of ethanol. In 96‐well plates, 300 μL of ABTS solution and 3 μL of culture medium (sample) were used per well. The absorbances for the ABTS and DPPH assays were measured at 630 nm and 490 nm, respectively, for a total of 10 and 60 min of incubation. As a negative standard, all solutions, that is, ABTS and DPPH diluted with their own solvent, as well as uncultured IVM medium containing ABTS and DPPH solutions were used. Finally, the antioxidant activity (AA) was expressed as the percentage of inhibition determined by the equation AA% = (AC—AS) / AC × 100, where AA (%) represents the percentage of antioxidant activity, AC represents the absorbance of the negative control and AS represents the absorbance of the sample. The average inhibitory concentration (IC50; mg/mL) was obtained through calibration curves, collected by plotting the different concentrations of AA% and later analysed by linear regression.

### 2.8 Statistical Analysis

Statistical analyses were performed using GraphPad Prism (version 7, Graphpad Software Inc., San Diego, CA, USA). Initially, the Shapiro–Wilk normality test was performed. Oocyte maturation rates were assessed by one‐way ANOVA and chi‐square test. The Kruskal–Wallis test was used to analyse enzyme activity (SOD, CAT) and thiol content, and the Tukey test was used for antioxidant capacity (DPPH and ABTS). Data are presented as mean ± standard error of the mean (s.e.m.) and percentage. Statistical significance was defined as *p* < 0.05.

## 3 Results

### 3.1 Evaluation of Oocyte Nuclear Maturation and Viability Rates

As shown in Table 1, AN300 reduced oocyte degeneration compared to AN30 (*p* < 0.05), with no differences in GV rates among treatments (*p* > 0.05). The AN2000 group showed higher GVBD rates than AN300 (*p* < 0.05), while AN300 increased MI rates relative to all other treatments (*p* < 0.05). Additionally, MII rates were higher in AN300 compared to AN2000 (*p* < 0.05). Overall, meiotic resumption (GVBD + MI + MII) was greatest in AN300 (*p* < 0.05).

**TABLE 1:** Percentage of degenerated (DEG), germinal vesicle (VG), germinal vesicle breakdown (GVBD), metaphase I (MI), or metaphase II (MII) oocytes, and meiotic resumption (MR) after IVM of ovine oocytes in the absence (CTRL) or presence of anethole at 30 (AN30), 300 (AN300), or 2000 (AN2000) μg/mL.

Treatments	DEG (%)	GV (%)	GVBD (%)	MI (%)	MII (%)	MR (%)
CTRL	22 (11/50)^A,B^	16 (8/50)^A^	10 (5/50)^A,B^	18 (9/50)^B^	34 (17/50)^A,B^	62 (31/50)^B^
AN30	24 (12/50)^A^	16 (8/50)^A^	12 (6/50)^A,B^	24 (12/50)^B^	24 (12/50)^A,B^	60 (30/50)^B^
AN300	6 (3/50)^B^	8 (4/50)^A^	6 (3/50)^B^	44 (22/50)^A^	36 (18/50)^A^	86 (43/50)^A^
AN2000	16 (8/50)^A,B^	20 (10/50)^A^	26 (13/50)^A^	22 (11/50)^B^	16 (8/50)^B^	64 (32/50)^B^

^A,B^ Different letters indicate significant differences within the same column (*p* < 0.05).

### 3.2 Intracellular ROS Levels and REDOX Balance in IVM Medium

**FIGURE 1:**
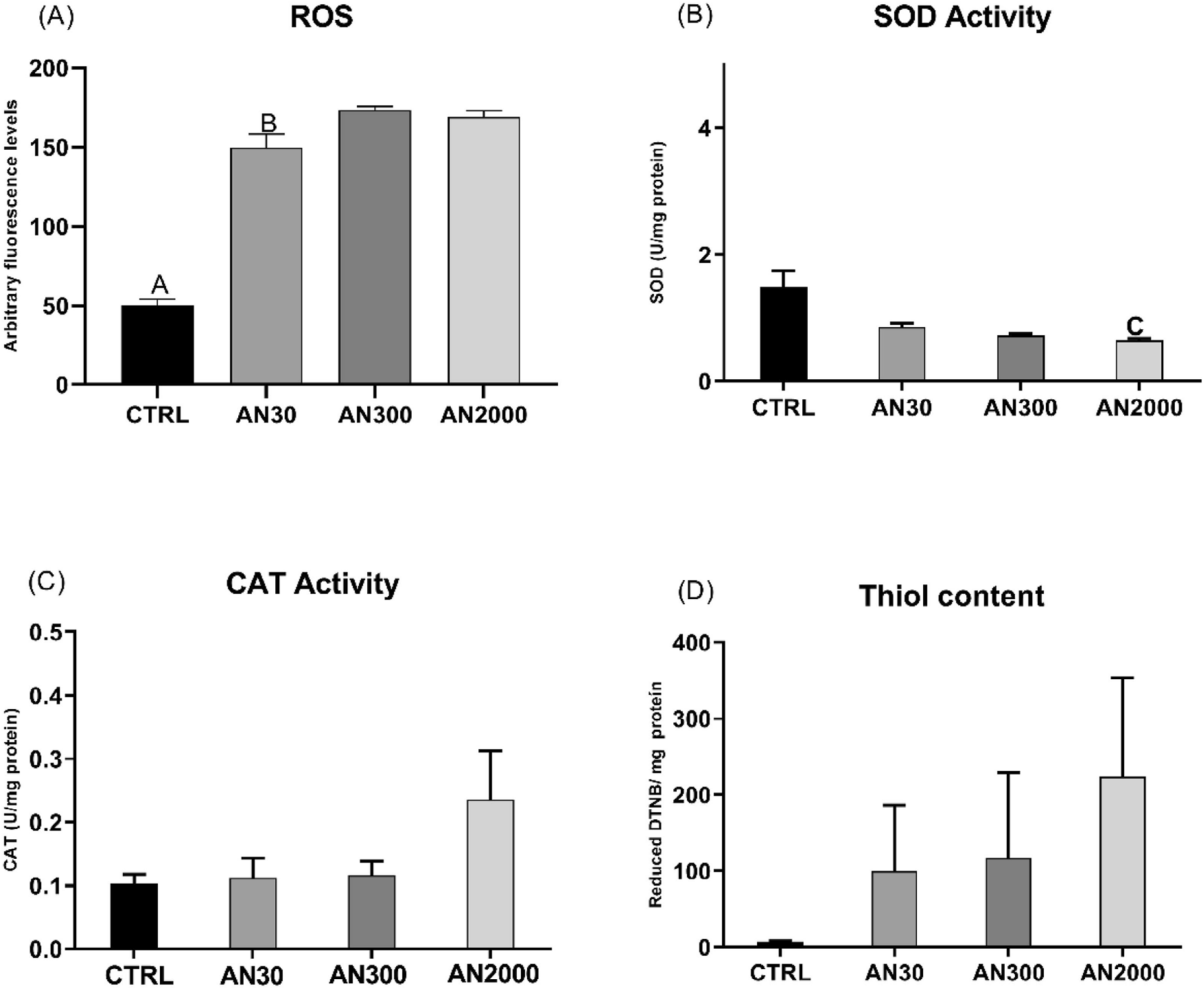
Redox status in oocytes and maturation medium after IVM of ovine oocytes in the absence (CTRL) or presence of anethole at different concentrations. (A) Mean intracellular ROS fluorescence intensity. SOD (B) and CAT (C) activity, and thiol content (D) in the IVM medium. ^AC^Indicate significant differences between treatments (*p* < 0.05).

Intracellular ROS levels after IVM were higher in the anethole treatments (Figure 1A), especially in AN300 and AN2000 (*p* < 0.05).

Data regarding antioxidant enzyme activity (SOD and CAT) and pro‐oxidant status (thiol content), evaluated in the maturation medium, are shown in Figure 1B–D, respectively. The presence of AN2000 reduced SOD activity compared to the other treatments (*p* < 0.05). However, regardless of concentration, anethole did not affect CAT activity or thiol content (*p* > 0.05).

### 3.3 Anethole Antioxidant Capacity

**FIGURE 2:**
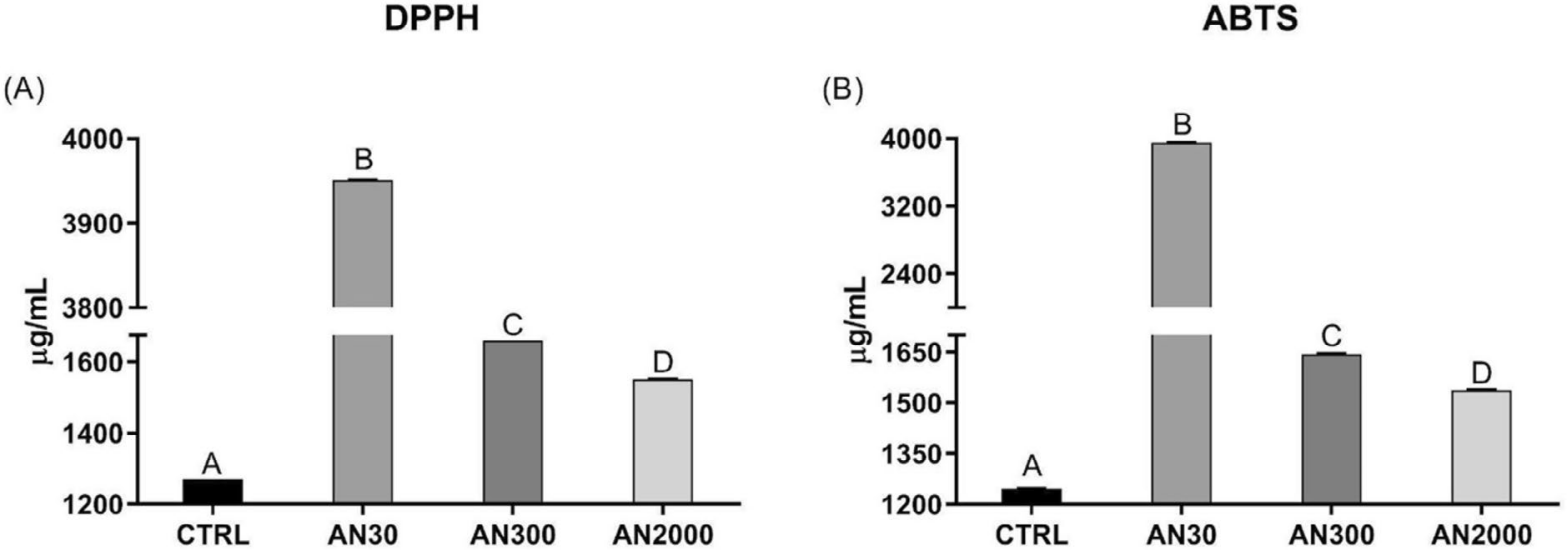
Assessment of the antioxidant capacity of different concentrations of anethole using different methodologies: (A) DPPH radical scavenging method, and (B) ABTS radical capture method. ^AD^Indicate significant differences between treatments (*p* < 0.05).

Figure 2 demonstrates the evaluation of the capacity of anethole to reduce free radicals by the DPPH (Figure 2A) and ABTS (Figure 2B) methods. In both tests, AN2000 was the anethole treatment that most reduced DPPH and ABTS, although it was lower than the control group (*p* < 0.05).

## 4 Discussion

This study evaluated for the first time the influence of anethole on the in vitro maturation of ovine oocytes. After IVM, anethole did not promote a higher rate of oocytes reaching metaphase II, although AN300 promoted a higher rate of meiotic resumption (GVBD + MI + MII) compared to the other treatments. Corroborating our findings, other natural antioxidants besides anethole, such as vitamin E (Natarajan et al. 2010), L‐carnitine (Mishra et al. 2016), resveratrol (Zabihi et al. 2019), and vitamin C (Tripathi et al. 2023), did not improve the rate of oocytes reaching the MII stage in sheep after IVM.

Reports on the use of anethole in IVM in other ruminants are conflicting: while in goats the beneficial effects of anethole on nuclear maturation (MII) were concentration‐dependent (Conceição‐Santos et al. 2024); in cattle, regardless of the concentration, there were no effects (Anjos et al. 2019; Sá et al. 2019; Janini et al. 2023). Chowdhury et al. (2018) reported that the use of antioxidants in IVM did not appear to influence nuclear maturation, but exerted beneficial effects on cytoplasmic maturation, which were reflected in subsequent early embryonic development. Therefore, it is interesting to investigate the subsequent impacts of anethole on early embryonic development in vitro in sheep. These effects can be explained by the fact that antioxidants can increase cellular glutathione content, a fact already demonstrated for anethole (Giustarini et al. 2020), which does not directly impact nuclear maturation, but acts by reducing the levels of ROS and pro‐apoptotic factors (Khazaei and Aghaz 2017). Supporting this, it was observed that 300 μg/mL of anethole added to the oocyte maturation medium, despite not affecting the rates of oocytes in MII, provided higher rates of cleavage and bovine blastocyst formation (Anjos et al. 2019; Janini et al. 2023).

During IVM, oocytes are sensitive to oxidative stress due to differences between the in vivo and in vitro environments (Khazaei and Aghaz 2017). Therefore, ROS‐induced oxidative stress causes a series of cellular consequences, such as lipid peroxidation, DNA and protein damage (Mauchart et al. 2023). Therefore, supplementing the IVM medium with antioxidants is necessary to prevent ROS accumulation and may improve cellular competence. Although the use of anethole as an antioxidant has shown beneficial effects on the female reproductive system of different species (Sá et al. 2019; Joo et al. 2023; Conceição‐Santos et al. 2024), its role in ovine IVM has not been previously demonstrated. In the present study, although all anethole concentrations tested increased intracellular ROS levels, this effect was not deleterious to oocytes, since oocyte viability was maintained. In agreement with our findings, Sá et al. (2019), Janini et al. (2023), and Conceição‐Santos et al. (2024) observed that the increase in ROS production by anethole stimulates mitochondrial activity, without influencing the redox status. Furthermore, it has been shown that the increase in ROS levels is related to increased metabolic activity, being beneficial for triggering the resumption of meiosis and stimulating the extrusion of the first polar body, and is not necessarily indicative of oxidative stress (Pandey et al. 2010). In addition, the follicular fluid naturally maintains a tightly regulated balance between reactive oxygen species and antioxidant defences, and this physiological redox environment is closely associated with oocyte competence and embryo production (Borjian‐Boroujeni et al. 2025). Therefore, the increased ROS observed in the present study, together with the maintenance of antioxidant defences and cell viability, suggests that anethole promoted a favourable redox regulation compatible with physiological signalling rather than harmful oxidative stress. Although the AN30 concentration presented lower ROS levels compared to the other anethole concentrations, this did not result in a significant increase in MII rates. Similarly, Sá et al. (2018), during in situ culture of follicles, observed that the AN30 concentration also maintained lower ROS levels; however, it was not able to sustain the percentage of morphologically normal follicles. These findings are consistent with our results and support the hypothesis that a reduction in ROS levels is not necessarily associated with improved cellular developmental competence; in many cases, increased cellular activity may lead to a transient imbalance, reflecting elevated metabolic demand during active cell development.

Regarding the activity of antioxidant enzymes, it was observed that the AN2000 treatment reduced SOD activity compared to the other treatments. However, regardless of the concentration, anethole did not affect CAT activity. During ROS formation, O_2_^−^, generated by oxidative metabolism in mitochondria and under the action of the enzyme SOD, is converted to H_2_O_2_ (Buettner 2011), which is then decomposed into water and oxygen by CAT (Khan et al. 2015). Giustarini et al. (2020) reported that anethole increases the activity of the antioxidants SOD and glutathione, acting to combat ROS, although this was not observed in our study. Our findings suggest that the increase in ROS in anethole treatments may not be related to oxidative stress, since there were no changes in the activities of the antioxidant enzymes evaluated. Furthermore, the maintenance of thiol content in the treated groups, compared to the control treatment, reinforces this hypothesis. Proteins with thiol groups (‐SH), such as GSH, require H_2_O_2_ to support disulfide bond (‐SS) formation (Foyer et al. 1997). The –SH group, present in GSH, can be used as an index of the oxidizing environment of a medium, since GSH oxidation by GPx only occurs in the presence of H_2_O_2_ (Furtado et al. 2021). Therefore, the thiol content can be interpreted as inversely proportional to the ROS (H_2_O_2_) content in a sample (Furtado et al. 2021).

The different concentrations of anethole used in this study were also subjected to DPPH and ABTS tests, in which it was observed that the antioxidant capacity of anethole in all treatments was lower than that of the control group. Nevertheless, it is important to highlight that anethole appears to demonstrate antioxidant activity in a concentration‐dependent manner, since AN2000 showed higher antioxidant capacity than AN30 and AN300. Both assays evaluate the ability of an antioxidant substance to neutralize ABTS and DPPH free radicals; therefore, the free radical content after the reaction is inversely proportional to the antioxidant capacity of the test substance. The findings of this study differ from those observed by Sá et al. (2019), in which anethole concentrations of 300 μg/mL showed greater antioxidant capacity, through the FRAP assay, compared to ascorbic acid. Sharopov et al. (2015) also observed that anethole showed good antioxidant activity in the ABTS and FRAP tests. However, in the DPPH test, its antioxidant activity was low. Similarly, Freire et al. (2005) observed little antioxidant activity for anethole. These differences can be explained by the fact that antioxidant capacity depends on the free radical evaluated and how the respective radical interacts with the antioxidant substance (Nie et al. 2021). Furthermore, variables such as reaction time mean that meaningful comparisons between the DPPH or ABTS methods are only possible when performed under the same conditions (Amorati et al. 2013), leading to conflicting findings regarding the antioxidant capacity of anethole and requiring further studies. Consequently, the decision was taken to evaluate both DPPH and ABTS in order to ensure the validity of our research findings.

In conclusion, the effects of anethole during in vitro maturation appear to be dose‐dependent and not exclusively associated with its antioxidant properties. Although reductions in oxidative markers were not consistently linked to improved outcomes, the AN300 concentration enhanced meiotic resumption, suggesting a potential beneficial effect on oocyte developmental competence. These findings indicate that optimal oocyte performance may depend on balance between oxidative regulation and other cellular mechanisms. However, future studies employing more specific markers of oxidative damage and cellular function are necessary to further elucidate the mechanisms underlying the observed effects. In addition, studies with larger experimental sample sizes would be valuable to confirm the effects of anethole on meiotic resumption observed in the present study. Therefore, anethole supplementation at an appropriate concentration may represent a promising strategy to improve IVM conditions, with potential implications for subsequent embryo production in ovine species, warranting further investigation.

## Author Contributions

Anderson Nascimento de Andrade, Naiza Arcangela Ribeiro de Sá and Valdevane Rocha Araújo designed the study. Anderson Nascimento de Andrade, Solano Dantas Martins, and Daniela Ribeiro Alves carried out the experimental procedures. Solano Dantas Martins, Alesandro Silva Ferreira, and Jonathan Elias Rodrigues Martins analysed the data. Anderson Nascimento de Andrade, Solano Dantas Martins, Alesandro Silva Ferreira, Luciana Rocha Faustino, and Valdevane Rocha Araújo wrote, edited, and revised the manuscript. Vânia Marilande Cecatto, José Henrique Leal‐Cardoso and Selene Maia de Morais provided the laboratory infrastructure to carry out experiments.

## Funding

This work was supported by Conselho Nacional de Desenvolvimento Científico e Tecnológico, 151664/2020‐0, and by the Research Incentive Program from Parnaíba Delta Federal University (UFDPar), under the call number 01/2024/PROPOPI/UFDPar.

## Ethics Statement

This study was approved and performed under the guidelines of the Ethics Committee for Animal Use of the State University of Ceará under protocol number 03821216/2021.

## Conflicts of Interest

The authors declare no conflicts of interest.

## Data Availability

The data that support the findings of this study are available from the corresponding author upon reasonable request.
